# A pilot randomized controlled trial to improve geriatric frailty

**DOI:** 10.1186/1471-2318-12-58

**Published:** 2012-09-25

**Authors:** Ding-Cheng Derrick Chan, Hsiao-Hui Tsou, Rong-Sen Yang, Jau-Yih Tsauo, Ching-Yu Chen, Chao Agnes Hsiung, Ken N Kuo

**Affiliations:** 1 Department of Geriatrics and Gerontology, and Department of Internal Medicine, National Taiwan University Hospital, No.7, ChongSan S. Rd, Taipei, Taiwan; 2 Department of Orthopaedics, National Taiwan University Hospital, No.7, ChongSan S. Rd, Taipei, Taiwan; 3 School and Graduate Institute of Phsical Therapy, College of Medicine, National Taiwan University, Taipei, Taiwan. Institution mailing address, Floor 3, No. 17, Xuzhou Rd, Taipei, Taiwan; 4 Department of Family Medicine, National Taiwan University Hospital, Taipei, No 7 ChungSan S. Rd, Taipei, Taiwan; 5 Institute of Population Health Sciences, National Health Research Institutes, R440, 4 F, No. 17 Xu-Zhou Road, Taipei, Taiwan; 6 Division of Biostatistics and Bioinformatics, Institute of Population Health Sciences, National Health Research Institutes, 35 Keyan Road, Zhunan, Miaoli County, Taiwan; 7 Institute of Population Health Sciences, National Health Research Institutes, 35 Keyan Road, Zhunan, Miaoli County, Taiwan

**Keywords:** Frailty, Aged, Intervention, Effectiveness, Community

## Abstract

**Background:**

Few randomized controlled trials (RCTs) report interventions targeting improvement of frailty status as an outcome.

**Methods:**

This RCT enrolled 117 older adults (65-79 years of age) in Toufen, Taiwan who scored 3-6 on The Chinese Canadian Study of Health and Aging Clinical Frailty Scale Telephone Version and then score ≥1 on the Cardiovascular Health Study Phenotypic Classification of Frailty (CHS_PCF). With a two by two factorial design, subjects were randomly assigned to interventions (Exercise and nutrition, EN, n = 55 or problem solving therapy, PST, n = 57) or controls (non-EN, n = 62 or non-PST, n = 60). Educational booklets were provided to all. EN group subjects received nutrition consultation and a thrice-weekly exercise-training program while PST group subjects received 6 sessions in 3 month. Subjects were followed at 3, 6, and 12 months. Primary outcome was improvement of the CHS_PCF by at least one category (from pre-frail to robust, or from frail to pre-frail or robust) from baseline assessments. One hundred and one completed final assessments. Intention-to-treat analysis with the generalized estimating equation model was applied with adjustment for time and treatment-by-time interactions.

**Results:**

Mean age was 71.4 ± 3.7 years, with 59% females. Baseline characteristic were generally comparable between groups. EN group subjects had a higher improvement rate on the primary outcome than non-EN group subjects (45% vs 27%, adjusted p = 0.008) at 3 months, but not 6 or 12 months. They also had more increase of serum 25(OH) vitamin D level (4.9 ± 7.7 vs 1.2 ± 5.4, p = 0.006) and lower percentage of osteopenia (74% vs 89% p = 0.042) at 12 months. PST group subjects had better improvement (2.7 ± 6.1 vs 0.2 ± 6.7, p = 0.035, 6-month) and less deterioration (−3.5 ± 9.7 vs −7.1 ± 8.7, p = 0.036, 12-month) of dominant leg extension power than non-PST subjects. Some secondary outcomes were also improved in control groups (non-EN or non-PST). No adverse effects were reported.

**Conclusions:**

The three-month EN intervention resulted in short-term (3-month) frailty status improvement and long-term effect on bone mineral density and serum vitamin D (12-month) among Taiwanese community-dwelling elders. The effect of PST was less pronounce.

**Trial registration:**

ClinicalTrials.gov: EC0970301

## Background

Frailty is a geriatric condition characterized by loss of reserves (energy, physical ability, cognition, health) that gives rise to vulnerability [1]. The lack of a consensus, however, on the definitions of and measurements for this geriatric condition has limited comparisons on the effectiveness of interventional studies on frail older adults [2]. Numerous instruments were developed to measure frailty. A recent review of on frailty instruments as outcome measures found that instruments could generally fit into 3 dimensions (physical, psychological, and social) with 8 factors (nutritional status, physical activity, mobility, energy, strength, cognition, mood, and social relationship/social support) [3]. However, it is not clear whether these instruments had sound clinimetric properties to be considered as good outcome measures that were responsive to interventions [3]. Another recent review on exercise interventions for management of frailty also pointed out that even all 47 studied enrolled “frail” older adults, validated operationalizations of frailty were only available for 3 studies [4]. None of the studies reviewed used frailty status as an outcome measure [4]. When we conducted a systemic review of frailty intervention focusing on trials that measured outcomes based on their pre-defined frailty indicators, only 11 studies were included [5]. Of the 4 studies [2,6-8] that enrolled participants based on the Cardiovascular Health Study Phenotypic Classification of Frailty (CHS_PCF) [9], one have not published their study outcome [2], and the rests [6-8] were not able to demonstrate the effects of interventions on indicators from the CHS_PCF.

Frailty has multiple etiologies, interacting pathogeneses, and often linked with other geriatric conditions and poor outcomes [10,11]. For example, a recent review found consistent bidirectional associations between depression and frailty in cross-sectional studies, but less consistent associations in cohort studies [12]. Similarly, osteoporosis and frailty shared many common risk factors — such as malnutrition, sarcopenia, physical inactivity, and low vitamin D [4,13-15] — that would increase the risk of fall and fracture [14].

However, it is not clear whether interventions targeting frailty or other geriatric conditions (eg: depression or osteoporosis) may benefit from each other.

We designed a pilot randomized control trail using validated frailty indicators to enroll 117 community-dwelling older adults with the following aims: 1) To determine whether the proposed interventions may have an impact on dynamic changes of frailty indicators. 2) To determine whether these interventions have impacts on other outcomes including depression, cognition, bone mineral density, physical function, and quality of life. 3) To explore the feasibility and accurate sample size to inform the design and implementation of future large scale clinical trial.

## Methods

**Figure 1:**
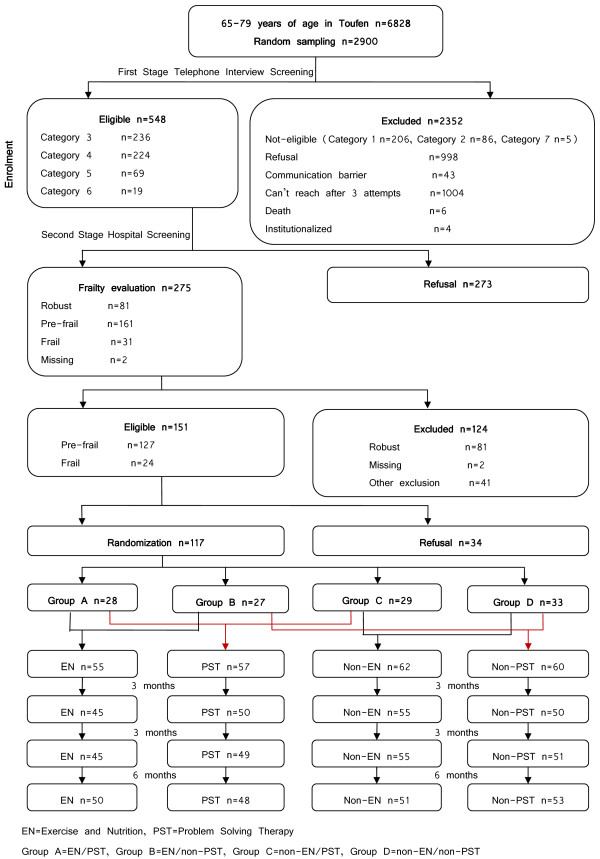
Flow chart.

A single site randomized controlled trial was conducted with a 3-month interventions and a 12-month follow-up period after baseline assessments on Taiwanese older adults with high frailty risk (Figure 1). The study was approved in 2008 by the Institutional Review Board of the National Health Research Institutes (NHRI), Zhunan, Taiwan.

### Recruitment and eligibility

Participants were enrolled after a telephone interview screening followed by a hospital screening. Our target population was community-dwelling older adults from 65 to 79 years of age in Toufen Township (N = 6,828). The Chinese Canadian Study of Health and Aging Clinical Frailty Scale Telephone Version (CCSHA_CFS_TV) [1,16] with satisfactory inter-rater reliability and criterion validity was used for the first stage screening. The instrument was particularly useful in population base screening for its short administration time (<3 min) and its easy implementation even by interviewers without formal training in geriatric care [16]. Eligible participants were those scored 3-6 on the CCSHA_CFS_TV. Exclusion criteria included institutionalizations; communication barriers; and scores of 1, 2, (too healthy) or 7 (too ill) on the CCSHA_CFS_TV.

Eligible older adults were invited to a local community hospital for second-stage screening during their annual geriatric health exams that included history and physical, blood works such as complete blood counts and blood chemistry. Informed consents were signed after careful explanations of the benefits and risks of proposed study. Participants were also asked to give permissions to use information gathered from the geriatric health exams as well as other blood works (such as 25(OH) vitamin D), or special tests (such as bone mineral density) needed from the study.

The CHS_PCF was used to select eligible participants [9]. Most cut-points were adapted from the CHS (Please refer to Table 1 for detail). Important modifications were:

**Table 1:** The modified Cardiovascular Health Study_ Phenotypic Classification of Frailty (CHS_PCF) criteria

**Characteristics**	**Definition**
Weight loss	Unintentional weight loss of more than 3 kg, or greater than 5% of body weight of the previous year
Exhaustion	Based on the Center for Epidemiologic Studies Depression Scale (CES-D). Self-report of either of: “I felt that everything I did was an effort” and “I could not get going.” were at least occasionally or more frequent
Low activity level	Based on the Taiwan International Physical Activity Questionnaire Short Form (IPAQ-SF). Weekly energy expenditure for activities ≧ 2 metabolic equivalent tasks (METs) of fewer than 383 kcal for men and 270 kcal for women
Slowness	Five-meter walking time, by gender and height:
	Men: time ≧7 s for height ≦ 173 cm or time ≧ 6 s for height > 173 cm
	Women: time ≧ 7 s for height ≦ 159 cm or time ≧ 6 s for height > 159 cm
Weakness	Grip strength (kg), 3 measurements of the dominant hand from a dynamometer (North Coast Medical Precision Instrument, NC70142), stratified by gender and body mass index (BMI) quartiles:
	Men: ≦ 29 kg for BMI ≦ 24 kg/m^2^, ≦30 kg for BMI 24.1-26 kg/m^2^, ≦30 kg for BMI 26.1-28 kg/m^2^, ≦32 kg for BMI > 28 kg/m^2^
	Women: ≦ 17 kg for BMI ≦ 23 kg/m^2^, ≦ 17.3 kg for BMI 23.1-26 kg/m^2^, ≦18 kg for BMI 26.1-29 kg/m^2^, ≦21 kg for BMI > 29 kg/m^2^
Overall frailty status	Robust: 0 indicator was present. Pre-frail: 1 or 2 indicators were present. Frail: ≧ 3 indicators were present

Weight loss of 3 kg (instead of 5 kg) was used to adjust for smaller body size for an East Asian population. The Taiwan IPAQ-SF (International Physical Activity Questionnaire Short Form) [17] instead of the Minnesota Leisure Time Physical Activity Questionnaire [18] was used to measure energy expenditure because the former has been validated in Taiwanese populations [17].

Exclusion criteria included hearing/visual impairments affecting daily activity; cognitive impairment, as defined by a Mini-Mental Status Exam (MMSE) score of ≤16 [19]; functional impairment, as defined by a Barthel Index (BI) ≤35 [20]; active alcohol-abuse problems, organic mental disorders; history of schizophrenia or a diagnosis of a bipolar disorder; any mental problems (other than depression) under psychiatric care; active suicidal ideation; and a score of 0 on the CHS_PCF [9].

### Measurements and procedures

Baseline assessments were completed before randomizations. Outcomes were assessed at the end of intervention (roughly 3 months after baseline assessments), 6 months and 12 months after baseline assessments.

### Baseline assessments

Other than the frailty related characteristics collected from screening stages, comprehensive assessments were performed to collect data on several domains including 1) demographics, 2) health related characteristics, 3) body composition, and musculoskeletal system characteristics, and 4) blood works. Important primary and secondary outcomes were listed below.

### Primary and secondary outcomes

The primary outcome was improvement of CHS_PCF by at least one category (from pre-frail to robust, or from frail to pre-frail or robust) [9]. Secondary outcomes included interval changes of the following indicators between baseline and repeated assessments. We also categorized secondary outcomes into the above domains. In the frailty index domain, we included each of the 5 indicator from the CHS_PCF [9]. In the health-related characteristics domain, we included the Mini Mental Status Exam (MMSE) [19], Primary Care Evaluation of Mental Disorders (PRIME-MD) score [21], and the Barthel Index (BI) score [20], health care resource utilization, and EQ-5D^*TM*^[22]. In the complex body composition and musculoskeletal system domain, we included, body mass index (BMI), fat free mass (FFM) (Inbody 3.0®, as a substitute of lean body mass), lowest T score from either spine or hip bone mineral density (BMD, Norland Excell Bone Densitometer®), left one-leg-stand time, and dominant leg extension power. Finally, for all the blood chemistry we collected, we only reported 25(OH) Vitamin D level as the outcome for this manuscript. Most primary and secondary outcomes were repeated at all follow up visits except for MMSE, BMD, and 25(OH) Vitamin D (only repeated at 12-month). To improve the comparability of the baseline and 12-month 25(OH) Vitamin D samples, we defrosted samples collected at 2 different time-points to run the radioimmunoassay (RIA) on the same kit (DiaSorin®, Minnesota, U.S.A.).

### Randomization

Subjects were stratified by age (65-74, 75-79) and gender to achieve balance of baseline characteristics. Within each stratum, a permuted block (4 persons/block) randomization method was used to ensure balanced assignments. The randomization code was generated from the off-site statistical center with a computer random number generator. Random group allocation was managed by a project manager not involved in assessment or intervention. In a 2 × 2 factorial design, subjects were first randomly assigned to an exercise and nutritional program (EN) or non-EN group. Within each group, subjects were further randomized to a problem solving therapy (PST) [23] group or non-PST group. Previous studies have shown that when PST was used, both mental and physical health were improved [23]. The 2 × 2 factorial design would help to determine the individual effect of two interventions on frailty.

### Blinding

The research assistants who performed baseline and outcome assessments were blinded from the randomization status. However, blinding to the intervention research assistants or participants was not possible with the designed interventions.

### Interventions

#### Education booklet

The educational booklet on frailty, healthy diets, exercise protocols, and self-coping strategies was given to all participants. Subjects who were randomized into the non-EN, non-PST groups were contacted monthly to check on how much they had read the booklet and how well they had complied with the suggested diet and exercise protocols. For those randomized into EN or PST groups, similar questions were asked during their visits to the study sites for their designated programs

#### Exercise and nutritional program

The EN group subjects were invited to take a structured exercise course at the participating hospital 3 times a week for 3 months. Each section lasted about 1 h. The exercise program included 15 min warm up with 10 min brisk walks followed by gentle stretching of major joints (cervical and lumbar joints) and muscles (such as biceps, triceps, hamstrings, rectus femoris, gastrocnemius, soleus) for 5 repetitions each. Resistance training (20-30 min) with rubber band and bottled water (0.6-1 L) as weight for major muscles of upper and lower limbs with 10 to 15 repetitions for each (such as deltoids, biceps, triceps, hand grasp, hip and knee flexors and extensors, hip abductors, ankle plantar flexors and dorsiflexors). Postural control activities and balance training were also provided for 10 min by asking participants to perform tandem gaits and one leg standing with eyes open/close (up to ones’ ability), step up and down stairs, toe walking and heel walking. Finally a 5-min cool down session with gentle relaxation movements are done. The research team also inquired about the subjects’ dietary compliance and responded to their dietary questions during the exercise sessions.

#### The problem solving therapy

The PST group subjects received 6 sessions therapy by trained case managers. It is a brief form of evidence-based psychotherapy that was originally developed in Britain for use by medical professionals in primary care. It teaches people how to solve the “here-and-now” problems contributing to their mood-related conditions and helps increase their self-efficacy [23]. Previous studies have shown that when PST was used to manage mental problems, both mental and physical health were improved [23].

#### Approaches to analysis

Data were coded to permit blinding to group allocation during statistical analysis. All statistical analyses were conducted using SAS software, Version 9.1 (SAS Institute, Inc., Cary, NC). Analysis was conducted at baseline and at 3-month, 6-month, and 12-month follow-up assessments in accord with the “intention-to-treat” principle (ITT). Summary statistics, including mean and standard deviation, were provided for continuous variables, such as age, MMSE, PRIME-MD, etc. Frequencies and proportions were used to summarize discrete variables, such as CCSHA_CFS TV, CHS_PCF categorization, etc. Missing variables were excluded from analyses. Baseline characteristics were compared between two groups using *t*-test for continuous variables and chi-square test with Fisher’s exact test when appropriate for categorical variables.

Because of the factorial design, we tested for a possible interaction between the 2 interventions (EN and PST) for each reported outcome. If there was no interaction, we examine the independent effect of one intervention controlling for the effect of the other. If there was significant interaction, we would perform subgroup analysis to report the effect of one intervention with or without the other intervention.

In our study, the outcomes of interest (e.g., frailty improvement) were measured at several time points (baseline, the 3rd month, the 6th month, and the 12th month). For estimating the repeated measurements of the intervention effect, the generalized estimating equations (GEE) model was used to compare the between-group frailty improvement with adjustment for time and treatment-by-time interactions. GEE approach is an extension of generalized linear model (GLM) and provides a semi-parametric approach to repeated categorical response. The intervention effect can be reasonably estimated by using GEE even if the covariance structure is not specified correctly. The primary outcome was also adjusted for multiple baseline characteristics, including age, gender, MMSE, healthcare-resource utilizations, EQ-5D, FFM, BMD, one-leg stand and 25 (OH) Vitamin D. Where high co-linearity was found between two potential confounders, only one variable was retained in the final model.

Longitudinal changes between groups and changes within a group were analyzed with the use of linear mixed models. However, the between-group BMD differences were compared using logistic regression model at 12 months. Under all circumstances, *P* < 0.05 was considered to be statistically significant.

## Results

### Participant flow

From the 6,828 target population, the probability-proportional-to-size (PPS) sampling approach was employed to select 2,900 population-representative random samples for first-stage screening. However, only 845 completed the telephone interview, 548 of whom were eligible for second-stage screening. Half (N = 275) signed the informed consents and were screened at the hospital. The recruitment period was roughly 3 months. Following the 2 by 2 factorial design protocol, 117 out of the 151 eligible subjects were randomized into 4 groups: A (EN/PST), B(EN/non-PST), C(non-EN/PST), and D(non-EN/non-PST). Group A and B were combined into the EN group (N = 55) while group C and D were combined into the non-EN group (N = 62). Similarly, Group A and C became the PST group (N = 57) while group B and D were the non-PST group (N = 60). Eighteen of the 55 EN group subjects attended at least 50% of the 36 intervention sessions while 16 of the 57 PST subjects completed the 6 courses. At 12 months, 50 (EN group), 51 (non-EN group), 48 (PST group), 53 (non-PST group) subjects completed final assessments, respectively. The reason for attrition was participant refusal (Figure 1).

### Baseline characteristics

For the entire cohort (N = 117), mean age was 71.4 ± 3.7; and 69 (59%) were female. Using the CCSHA_CFS TV, 47 (40%) were categorized into category 3 (well, with treated co-morbid diseases), 55 (47%) into category 4 (apparently vulnerable), 13 (11%) into category 5 (mildly frail), and 2 (2%) into category 6 (moderately frail). With the CHS_PCF, 102 (87%) were classified pre-frail, and 15 (13%) as frail at baseline.

Even though all subjects were considered at high risk for frailty, subjects enrolled in the trial were relatively healthy with few co-morbidities (3.5 ± 2.2), preserved BI (mean 98.3 ± 4.7) score, MMSE (24.4 ± 3.9) scores, low PRIME-MD (2.5 ± 3.4) score, satisfactory EQ-5D score (0.9 ± 0.1), and few healthcare-resource utilizations (1.6 ± 2.0). The cohort had high prevalence of radiographic vertebral fracture (N = 111, 95%), and high prevalence of osteopenia (n = 92, 80%) from DXA scan. The mean left one leg stand time was 5.8 ± 6.3 s, the mean dominant leg extension power is 25.7 ± 6.1 kg, and the mean 25 (OH) Vitamin D level was 17.5 ± 5.8 (ng/ml).

Most baseline characteristics were similar between EN and no-EN group, also between PST, and non-PST group (Table 2). However, EN group subjects had lower percentage of weakness (60% vs 81%, p = 0.014), higher number of co-morbid conditions (4.0 ± 2.1 vs 3.1 ± 2.2, p = 0.022) (Table 2). Also, PST group subjects higher percentage of slowness (26% vs 12%, p = 0.043).

**Table 2:** Baseline Characteristics of the Participants, Total Patient Number = 117

**Characteristics**	**EN (N = 55)**	**Non-EN (N = 62)**	**P-value**‡	**PST (N = 57)**	**Non-PST (N = 60)**	**P-value**‡
	**n (%) mean ± sd**†	**n (%) mean ± sd**†		**n (%) mean ± sd**†	**n (%) mean ± sd**†	
**Frailty-Related Characteristics**						
CCSHA_CFS TV						
well, with treated co-morbid diseases (3)	24 (44)	23 (37 )	0.719	24 (42)	23 (38)	0.755
apparently vulnerable (4)	25 (45)	30 (48)		27 (47)	28 (7)	
mildly frail (5) + moderately frail (6)	6 (11)	9 (15)		6 (11)	9 (15)	
CHS_PCF categorization						
pre-frail (1–2)	46 (84)	56 (90)	0.280	48 (84)	54 (90)	0.349
frail (3–5)	9 (16)	6 (10)		9 (16 )	6 (10 )	
CHS_PCF characteristics						
Weight loss (yes)	18 (33)	12 (19)	0.098	12 (21 )	18 (3)	0.268
Exhaustion (yes)	25 (45 )	23 (37)	0.359	22 (39)	26(43)	0.603
Low activity level (yes)	3 (5)	6 (10)	0.498	5(9)	4(7)	0.739
Slowness (yes)	10(18)	12(9)	0.871	15 (26)	7 (12)	0.043
Weakness (yes)	33 (60)	50 (81)	0.014	42 (74)	41 (68)	0.524
**Demographics**						
Age (y/o)	70.9 ± 3.7	71.9 ± 3.7	0.158	71.5 ±3.7	71.3 ± 3.9	0.673
Female sex	33(60)	36(58)	0.832	33(58)	36 (60)	0.817
**Health-Related Characteristics**						
Number of chronic conditions^§^(N = 114)	4.0 ± 2.1	3.1 ± 2.2	0.022	3.8 ± 2.4	3.2 ± 1.9	0.184
MMSE	24.8 ± 3.9	24.1 ± 3.9	0.358	24.7 ± 3.8	24.2 ± 4.0	0.552
PRIME-MD	2.1 ± 3.2	2.8 ± 3.5	0.229	2.7 ± 3.3	2.3 ± 3.4	0.469
Barthel Index	98.8 ± 3.7	97.9 ± 5.4	0.284	98.2 ± 5.4	98.4 ± 4.0	0.846
EQ-5D	0.94 ± 0.08	0.94 ± 0.08	0.969	0.95 ± 0.08	0.93 ± 0.08	0.246
Healthcare-resource utilization	1.5 ± 1.7	1.7 ± 2.2	0.517	1.6 ± 1.7	1.7 ± 2.2	0.846
**Body Composition, and Musculoskeletal System Characteristics**						
BMI (kg/m^2^)	25.0 ± 3.3	25.8 ± 3.9	0.229	25.0 ± 3.8	25.8 ± 3.5	0.251
FFM (kg)	42.3 ± 7.0	43.6 ± 7. 9	0.345	42.2 ± 7.3	43.7 ± 7.6	0.275
Compres sion Fracture form Spine XRAY	53 (96)	58 (94)	0.683	54 (95)	57 (95)	1.000
BMD (T-score)^∥^ (N = 115)						
> −1	13 (25)	10(16)	0.262	13 (23)	10(17)	0.401
≦ −1	40 (75)	52 (84)		43 (77)	49 (83)	
Left one leg stand time (sec) (N = 111)	5.7 ± 6.9	5.8 ± 5.9	0.949	5.3 ± 6.7	6.2 ± 6.0	0.483
Dominant leg extension power (kg)	26.3 ± 5.1	25.2 ± 6.8	0.295	23.9 ± 6.5	27.4 ± 5.1	0.002
**Blood Examination**						
25(OH) Vitamin D (ng/mL) (N = 109)	17.8 ± 5.3	17.2 ± 6.2	0.558	17.9 ± 5.5	17.2 ± 6.0	0.514

†:Categorical data:n (%);Continuous variables:mean **±** sd.

‡:Categorical data:*χ*^2^ test or Fisher’s exact test;Continuous variables: *t*-test.

^§^:From 27 diseases.

^¶^:Emergency room visits, hospitalizations, or clinic visits in the past 3 months.

^∥^:The minimum T-score of either the femoral neck or the spinal mean at L2–L4.

*BMD*:Bone Mineral Density, *BMI*: Body mass index, *CCSHA_CFS TV*:Chinese Canadian Study of Health and Aging_Clinical Frailty Scale Telephone Version, *CHS_PCF*:Cardiovascular Health Study_Phenotypical Classifica tion of Frailty, *EN*:Exercise and Nutrition, *EQ*-5D:EuroQol Quality of Life Scale, *FFM*:Fat Free Mass, *MMSE*:Mini-Mental State Examination, *PRIME-MD*:Primary Care Evaluation of Mental Disorders, *PST*:Problem Solving Therapy.

### Primary outcomes and transition of frailty status

**Figure 2:**
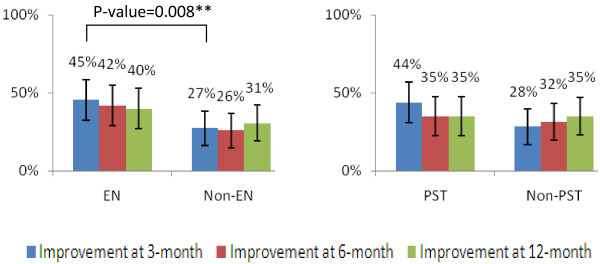
Primary Outcome.

The improvement rates were highest at the end of intervention (3-month) for EN (45%) and PST (44%) groups. Afterwards, there were gradual declines of the improvement rates at 6 (42% EN group, 35% PST group) and 12 (40%, EN group, 35% PST group) months. On the other hands, the improvement rates of the non-EN, or non-PST group subjects were stable around 30%. Therefore, only the 3-month differences between EN and non-EN group (45% vs 27% p = 0.008) was significant after adjusting the effect of PST and other potential confounders (Figure 2).

During the intervention period (baseline to 3-month), 33 (32.4%) of the prefrail participants (N = 102) improved to robust, while 3 (20%) and 6(40%) of the frail (N = 15) participants improved to robust and prefrail, respectively (Table 3). During follow-up periods without intensive interventions, most individual stayed at their original status, the chances for natural transition to better frailty status was a lot fewer. For example, during the 3-6 month follow up period, only 2 (22.2%) of frail individuals (N = 9) improved to prefail status, and only 12 (16.7%) of prefrail individuals (N = 72) improved to robust status.

**Table 3:** Transitions among frailty states at different time points

	**Baseline to 3 months**		**3 to 6 months**		**6 to 12 months**	
Transition	No.	Rate,%	No.	Rate,%	No.	Rate,%
Robust to	-	-	N = 36		N = 34	
Robust	-	-	22	61.1	25	73.5
Pre-frail	-	-	13	36.1	9	26.5
Frail	-	-	1	2.8	0	0.0
Pre-frail to	N = 102		N = 72		N = 69	
Robust	33	32.4	12	16.7	10	14.5
Pre-frail	66	64.7	54	75.0	58	84.1
Frail	3	2.9	6	8.3	1	1.4
Frail to	N = 15		N = 9		N = 14	
Robust	3	20.0	0	0.0	0	0.0
Pre-frail	6	40.0	2	22.2	3	21.4
Frail	6	40.0	7	77.8	11	78.6

### Secondary outcomes

#### Individual frailty indicator

In general, no within or between group differences were observed over repeated measures (Table 4).

**Table 4:** Follow-up analysis (Intent to treat), Total Patient Number = 117

**Characteristics**	**EN (N = 55)**	**Non-EN (N = 62)**	**p-value**‡	**PST (N = 57)**	**Non-PST (N = 60)**	**p-value**‡
	**n (%).mean ± sd†**	**n (%) mean ± sd†**		**n (%)mean ± sd†**	**n (%) mean ± sd†**	
**Frailty-related Characteristics**						
Improvement of CHS_PCF characteristics^§^						
Weight loss (yes)						
Improvement at 3-month	9 (16)	6 (10)	0.277	7 (12)	8 (13)	0.838
Improvement at 6-month	8 (15)	8 (13)	0.761	6 (11)	10 (17)	0.329
Improvement at 12-month	11 (20)	9 (15)	0.400	8 (14)	12 (20)	0.372
Exhaustion (yes)						
Improvement at 3-month	16 (29)	17 (27)	0.824	16 (28)	17(28)	0.968
Improvement at 6-month	17 (31)	18(29)	0.806	16 (28)	19 (42)	0.666
Improvement at 12-month	19 (35)	20 (32)	0.769	16 (28)	23 (38)	0.328
Low activity level (yes)						
Improvement at 3-month	2(4)	4 (6)	0.495	3(5)	3(5)	0.926
Improvement at 6-month	2 (4)	4 (6)	0.495	3 (5)	3 (5)	0.926
Improvement at 12-month	2(4)	4 (6)	0.495	3(5)	3 (5)	0.926
Slowness (yes)						
Improvement at 3-month	6 (11)	2 (3)	0.126	5 (9)	3 (5)	0.423
Improvement at 6-month	4 (7)	3 (5)	0.585	6 (11)	1 (2)	0.085
Improvement at 12-month	6 (11)	3 (5)	0.232	7 (12)	2 (3)	0.090
Weakness (yes)						
Improvement at 3-month	11 (20)	17 (27)	0.346	17 (30)	11 (18)	0.135
Improvement at 6-month	9 (16)	16 (26)	0.218	9 (16)	16 (27)	0.170
Improvement at 12-month	7 (13)	17 (27)	0.055	12 (21)	12 (20)	0.837
**Health-Related Characteristics**						
MMSE						
Change at 12-month	−0.15 ± 2.53	0.06 ± 2.52	0.658	−0.05 ± 2.35	−0.02 ± 2.69	0.954
PRIME-MD						
Change at 3-month	−0.96 ± 2.92	−1.29 ± 4.50**	0.631	−1.32 ± 3.64*	−0.97 ± 4.03	0.603
Change at 6-month	−0.05 ± 2.84	−0.65 ± 4.03	0.356	−0.42 ± 2.96	−0.32 ± 4.00	0.846
Change at 12-month	−0.16 ± 3.17	−0.77 ± 3.65	0.327	−0.77 ± 3.27	−0.22 ± 3.58	0.365
Barthel Index						
Change at 3-month	1.09 ± 3.81*	1.53 ± 4.11**	0.520	1.05 ± 3.98*	1.58 ± 3.96**	0.458
Change at 6-month	0.36 ± 2.33	0.73 ± 4.78	0.597	0.88 ± 4.13	0.25 ± 3.50	0.354
Change at 12-month	0.55 ± 2.99	0.89 ± 3.68	0.617	0.88 ± 3.29	0.58 ± 3.46	0.655
EQ-5D						
Change at 3-month	0.02 ± 0.08	0.03 ± 0.08*	0.632	0.01 ± 0.09	0.03 ± 0.08**	0.162
Change at 6-month	−0.004 ± 0.12	0.004 ± 0.12	0.738	0.0001 ± 0.09	0.001 ± 0.14	0.980
Change at 12-month	0.01 ± 0.09	0.02 ± 0.10	0.455	0.01 ± 0.07	0.02 ± 0.11	0.534
Healthcare-resource utilization						
Change at 3-month	0.04 ± 1.36	−0.35 ± 2.70	0.353	−0.07 ± 1.67	−0.27 ± 2.58	0.650
Change at 6-month	0.60 ± 1.81	0.03 ± 2.55	0.186	0.42 ± 1.74	0.18 ± 2.65	0.589
Change at 12-month	0.05 ± 1.8	0.03 ± 2.44	0.984	0.39 ± 2.05	−0.28 ± 2.25	0.104
**Body Composition, and Musculoskeletal System Characteristics**						
BMI (kg/m^2^)						
Change at 12-month	−0.31 ± 1.19*	−0.18 ± 1.05	0.572	−0.36 ± 1.15*	−0.13 ± 1.08	0.280
FFM (kg)						
Change at 12-month	−0.46 ± 1.36*	−0.62 ± 1.84**	0.587	−0.59 ± 1.30**	−0.50 ± 1.90*	0.726
Compression Fracture form Spine X-ray						
12-month	55(100)	58(94)	0.137	54 (95)	59(98)	0.518
BMD (T-score)^∥^ (N = 115)						
12-month						
> −1	14(26)	7(11)	0.042	11(20)	10(17)	0.770
≦ −1	39(74)	55(89)		45(80)	49 (83)	
Left one leg stand time (sec) (N = 111)						
Change at 3-month	2.86 ± 9.19*	0.92 ± 9.01	0.268	2.38 ± 8.91	1.34 ± 9.34	0.553
Change at 6-month	2.57 ± 8.39*	1.81 ± 8.47	0.683	3.10 ± 8.93*	1.30 ± 7.84	0.298
Change at 12-month	3.69 ± 9.15**	3.43 ±9.15**	0.906	4.31 ± 10.23***	2.84 ± 7.92*	0.399
Dominant leg extension power (kg)						
Change at 3-month	3.06 ± 7.13**	1.72 ± 6.6*	0.330	3.42 ± 7.36***	1.33 ± 6.32	0.102
Change at 6-month	1.48 ± 5.9	1.35 ± 7.00	0.986	2.71 ± 6.08**	0.18 ± 6.68	0.035
Change at 12-month	−6.44 ± 10.08***	−4.44 ± 8.59***	0.217	−3.52 ± 9.65**	−7.14 ± 8.74***	0.036
**Blood Examination**						
25(OH) Vitamin D (ng/mL) (N = 109)						
Change at 12-month	4.85 ± 7.69***	1.19 ± 5.41	0.006	3.4 ± 7.80***	2.49 ± 5.85**	0.633

*P-value < 0.05, ** p-value < 0.01, *** p-value < 0.001 for the comparison of the value at the follow-up time with the baseline value within the group, as calculated with the use of linear mixed model.

†:Categorical data:n (%);Continuous variables:mean **±** sd.

‡: Intervention effect, controlling for the other treatment strategy, follow up time and treatment-by-follow up time interactions. Categorical data:generalized estimating equations (*GEE*);Continuous variables:linear mixed model. P-values for compression fracture form spine XRAY and BMD from logistic regression model with exact modification when appropriate.

^§^:After intervention 3, 6 and 12 months[*CHS_PCF*]has progressed from[yes]to[no].

^¶^:Emergency room visits, hospitalizations, or clinic visits in the past 3 months.

^∥^:The minimum T-score of either the femoral neck or the spinal mean at L2–L4.

*BMD*:Bone Mineral Density, *BMI*: Body mass index, *CHS_PCF*: Cardiovascular Health Study_Phenotypical Classification of Frailty, *EN*:Exercise and Nutrition, *EQ*-5D:EuroQol Quality of Life Scale, *FFM*: Fat Free Mass, *MMSE*: Mini-Mental State Examination, *PRIME-MD*: Primary Care Evaluation of Mental Disorders, *PST*: Problem Solving Therapy.

#### Health-related characteristics

There were no observable between group changes. However, within group improvements were found for PRIME-MD (non-EN and PST group at 3-month), BI (all 4 groups at 3-month), and EQ-5D (non-EN and non-PST group at 3-month) (Table 4).

#### Body composition, and musculoskeletal system characteristics

In general, there were no observable between group differences except that changes of dominant leg extension power at 6 and 12 months were in favor of the PST group (both p < 0.05). At 12 month, BMI decreased in both EN and PST group, but not the non-EN or non-PST group. However, FFM decreased in all 4 groups. For one leg stand time and leg extension power, changes at different time periods were general more obvious in intervention (EN or PST) than control (non-EN, non-PST) groups (Table 4).

#### Blood test

Increase of 25 (OH) Vitamin D level was observed in EN, PST, and non-PST groups in 12 months. The difference between EN and non-EN group (4.9 ± 7.7 vs 1.2 ± 5.4, p = 0.006) was statistically significant (Table 4).

#### Interaction

Interactions between EN and PST were found for improvement of weight loss and 25 (OH) Vitamin D. Controlling the effect of PST, the effect of EN was more significant in EN/non-PST subgroup (weight loss), and EN/PST subgroup (25 (OH) Vitamin D)(Additional file 1: Table S1). Controlling for the effect of EN, the effect of PST was more significant in PST/EN subgroup (for both variables) (Additional file 2: Table S2).

## Discussion

Our study demonstrates that the three-month exercise and nutritional program resulted in short-term (3-month) frailty status improvement and long-term effect on BMD and serum 25 OH Vitamin D (12-month) among a population-representative sample of frail older adults. The effect of PST on geriatric frailty, mood, and physical performance was less pronounce. We also found some significant improvements in the control (non-EN, non-PST) groups.

Many instruments were created to measure frailty and studies with different instruments were difficult to compare with [24]. We chose several recent intervention trials that used modified CHS_PCF for comparison [2,6-8]. Peterson et al. enrolled 81 older male veterans scored ≥1 on CHS_PCF. Roughly half (N = 39) were randomized into a high intensity physical activity telephone counseling group [7]. After 6-months, 49% and 69% were still classified as frail respectively (p = 0.08). Kenny and colleague reported the effect of 12-month transdermal testosterone patch on 131 older men with low testosterone level, fracture, or low BMD and scored ≥1 on CHS_PCF [6]. Improvements of BMD, and lean mass were found, but not physical performance or frailty indicators [6]. In another RCT, Li et al. enrolled 310 community-dwelling older adults who scored ≥1 on the CHS_PCF [8]. The 6-month individualized multi-factorial care plans after comprehensive geriatric assessments (CGAs) did not improve frailty status. The Frailty Intervention Trial (FIT) [2] used similar CGA with individualized care plans approach but enrolled older adults with ≥3 deficiencies; that study is still in progress and so still lacks published outcome data. To our knowledge, ours is the first study to demonstrate that the CHS_PCF categorization is responsive to intervention to with sound clinimetric properties as an outcome measure [24].

When other frailty indicators were considered, several recent reviews found that structured exercise improved physical and psychological determinants, frailty status, and prevented disability in frail older adults [4,5,25,26]. However, many researchers called for more unified definition and operationalization of frailty to enhance comparability of different intervention trials [2,4,5,27].

Expert opinions and the results of clinical trials suggest nutritional consultation as a component in frailty interventions [2,28,29]; but rarely does it stand as an independent intervention on frailty. One recent study showed that diet and exercise was more effective than diet or exercise alone in improving frailty indicators among 93 obese and frail older adults [29]. Our study also added new evidence that combination of exercise program and nutritional information had positive impact on frailty.

It is not clear whether exercise and/or nutritional consultation has a positive impact on BMD or 25 (OH) Vitamin D level among frail older adults. In a study of 65 subjects randomized to moderate-intensity on-site exercise training 3 times per week for 9 months,, the subjects’ BMD did not differ from that of 47 subjects randomized to a low-intensity home exercise program [30]. Similar to our study, Villareal and colleagues reported positive effect of diet and exercise on improving or preserving BMD from 2 RCTs of 27 [31] and 93 [29] obese older adults. This research group [31] also found that diet and exercise increased the serum 25(OH) Vitamin D level as in our study.

In our study, subjects in the PST group had better improvement in frailty and PRIME-MD scores than subjects in the non-PST group; but the differences did not reach statistical significance. Even roughly 40% reported exhaustion from the (CES-D) [32] questions, their mean PRIME-MD score was quite low (average 2 points) indicating low level of depression. The floor effect might explain parts of the lack of effectiveness of PST.

Some observational studies suggested that frailty is a dynamic process and natural transitions to better status may occur without interventions [33-35]. During the intervention period, our degrees of improvement in frailty status were significantly higher than the natural improvement rates reported from observational studies [34]. On the other hand, the improvement rates during the follow up periods were similar to other studies [34]. It was encouraging that frailty status could be reversed with proper interventions. However, the effects might not last long when intensive interventions were discontinued.

We felt it unethical to enroll older adults in the control group without basic education material to teach them about self-managements even this might mitigate intervention effect. It is encouraging that subjects received education material only also had improvements in functional status, mood, quality of life, and physical performance.

### Strengths and limitations of the current study

The probability sampling design enhances the generalizability of this study to community-dwelling frail older adults without significant cognitive or functional impairments. The quick and valid CCSHA_CFS TV saved us tremendous time and resources in conducting the large-scale community-based frailty screening. Our educational material and interventions would be easily replicable in other settings.

The study also has several important limitations. First, we encountered an unexpectedly low response rate during the telephone-interview stage, with one-third not being reachable after multiple attempts, and another one-third refusing the telephone interviews, which hampers the external validity of the current study.

Second, compliance with the thrice-weekly exercise sessions and PST sessions were fair. Many participants had problems reaching the study site and other personal issues, such as taking care of their grandchildren which prevented them from on-site intervention. The intervention effect could have been enhanced if better adherence had been reached.

Third, the CHS_PCF instrument does not allow assessment of different degrees of frailty as the CCSHA_CFS_TV. However, we were not able to detect more subtle changes frailty degrees with the later instrument since it was only used at the screening stage.

Forth, we did not have use population specific cut-points in the 5 frailty indicators to enroll study participants. At the time of the study design, Taiwanese frailty cut-points with the CHS_PCF were not available. However, since it is an interventional study with a purpose to identify subjects with certain degree of frailty suitable for interventions, it probabably did not matter which cut-points were used as long as study populations could be clearly and systemicly identified and classified.

Finally, the study sample size is relatively small, though it is comparable or greater than some previous interventional studies of frailty [6,7,28-31]. In the review conducted by Thou and colleagues [4], only 13 out of 47 exercise programs had sample size greater than our study which indicating the difficulty enrolling and conducting RCTs in frail older adults. Since there was a lack of previous data to guide estimation of sample size based on our designated primary outcome, one purpose of this study was to determine feasible sample size for future study.

## Conclusions

In summary, with proper exercise and nutritional management even a short, 3-month intervention can improve the dynamic frailty process, bone mineral density, and 25(OH) Vitamin D level in frail older adults.

## Competing interests

All authors do not have competing interests.

## Authors’ contributions

All of the authors participated in the preparation of the manuscript. Specifically, D-C D C, R-S Y, and J-Y T wrote the first draft of the manuscript. H-H T and C A H performed statistical analysis and wrote the analysis section. C-Y C and K N K designed the study and provided substantial revisions to the manuscript. All authors have read and approved the final manuscript.

## Authors’ information

All authors had access to the data and had a role in writing the manuscript.

## Funding

This work was supported by the National Health Research Institutes, Zhunan, Taiwan grant (97-HD-SP-08 “Interventional Study of Geriatric Frailty, Osteoporosis, and Depression in a Community Based Randomized Trial”).

## Pre-publication history

The pre-publication history for this paper can be accessed here:

http://www.biomedcentral.com/1471-2318/12/58/prepub

## Supplementary Material

Additional file 1 **Table S1.** Follow-up analysis (Intent to treat), Total Patient Number=117. (Interactions between EN and PST were found for improvement of weight loss and 25 (OH) Vitamin D. Table 2s_1:EN vs. non-EN, controlling for PST or non-PST). Click here for file

Additional file 2 **Table S2.** Follow-up analysis (Intent to treat), Total Patient Number=117. (Interactions between EN and PST were found for improvement of weight loss and 25 (OH) Vitamin D. Table 2s_2:PST vs. non-PST, controlling for EN or non-EN). Click here for file
