# Supplementary material for: A pilot randomized controlled trial to improve geriatric frailty
Source: BMC Geriatr. 2012 Sep 25;12:58. doi: 10.1186/1471-2318-12-58 (PMC3490887; doi:10.1186/1471-2318-12-58)
Supplement: Additional file 2 — Table S2. Follow-up analysis (Intent to treat), Total Patient Number=117. (Interactions between EN and PST were found for improvement of weight loss and 25 (OH) Vitamin D. Table 2s_2:PST vs. non-PST, controlling for EN or non-EN). [file 1471-2318-12-58-S2.doc]

Appendix Table2 Follow-up analysis (Intent to treat), Total Patient Number=117

(Interactions between EN and PST were found for improvement of weight loss and 25 (OH) Vitamin D. Table 2s_2：PST vs. non-PST, controlling for EN or non-EN)

|  |  | **PST/EN**  **(N=28)** | | | |  | **Non-PST/EN (N=27)** | | | |  |  | **PST/Non-EN (N=29)** | | | | |  | **Non-PST/Non-EN (N=33)** | | | |  |  |
| --- | --- | --- | --- | --- | --- | --- | --- | --- | --- | --- | --- | --- | --- | --- | --- | --- | --- | --- | --- | --- | --- | --- | --- | --- |
|  |  | **n (%) mean ± sd**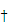 | | | |  | **n (%) mean ± sd**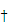 | | | |  | **P-value**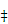 |  | **n (%) mean ± sd**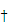 | | | |  | **n (%) mean ± sd**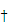 | | | |  | **P-value**^§^ |
| **Improvement of CHS_PCF characteristics**^¶^ |  |  |  |  |  |  |  |  |  |  |  |  |  |  |  |  |  |  |  |  |  |  |  |  |
| Weight loss (yes) |  |  |  |  |  |  |  |  |  |  |  |  |  |  |  |  |  |  |  |  |  |  |  |  |
| Improvement at 3-month |  | 2 | ( | 7 | ) |  | 7 | ( | 26 | ) |  | 0.152 |  | 5 | ( | 17 | ) |  | 1 | ( | 3 | ) |  | 0.177 |
| Improvement at 6-month |  | 1 | ( | 4 | ) |  | 7 | ( | 26 | ) |  | 0.028 |  | 5 | ( | 17 | ) |  | 3 | ( | 9 | ) |  | 0.532 |
| Improvement at 12-month |  | 3 | ( | 11 | ) |  | 8 | ( | 30 | ) |  | 0.050 |  | 5 | ( | 17 | ) |  | 4 | ( | 12 | ) |  | 0.412 |
| 25(OH) Vitamin D (ng/mL) (N=109) |  | (N=27) | | |  |  | (N=25) | | |  |  |  |  | (N=24) | | | |  | (N=33) | | | |  |  |
| Change at 12-month |  | 6.85 | ± | 7.88*** |  |  | 2.70 | ± | 7.01 | * |  | 0.022 |  | -0.37 | ± | 5.77 |  |  | 2.32 | ± | 4.91 | * |  | 0.120 |

*P-value < 0.05, ** p-value < 0.01, *** p-value < 0.001 for the comparison of the value at the follow-up time with the baseline value within the group, as calculated with the use of linear mixed model.


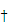
：Categorical data：n (%)；Continuous variables：mean ± sd.


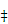
：PST vs. Non-PST, if EN. Categorical data：generalized estimating equations (GEE)；Continuous variables：linear mixed model.

^§^：PST vs. Non-PST, if Non-EN. Categorical data：generalized estimating equations (GEE)；Continuous variables：linear mixed model.

^¶^：After intervention 3, 6 and 12 months「CHS_PCF indicator」has progressed from「yes」to「no」.

CHS_PCF：Cardiovascular Health Study_Phenotypical Classification of Frailty, EN：Exercise and Nutritional, PST：Problem Solving Therapy
